# LVPocket: integrated 3D global-local information to protein binding pockets prediction with transfer learning of protein structure classification

**DOI:** 10.1186/s13321-024-00871-8

**Published:** 2024-07-07

**Authors:** Ruifeng Zhou, Jing Fan, Sishu Li, Wenjie Zeng, Yilun Chen, Xiaoshan Zheng, Hongyang Chen, Jun Liao

**Affiliations:** 1 School of Science, China Pharmaceutical University, Nanjing, 210009 Jiangsu People’s Republic of China; 2 Zhejiang Lab, Hangzhou, 311121 Zhejiang People’s Republic of China; 3 Research Center for Graph Computing, Zhejiang Lab, Hangzhou, 311121 Zhejiang People’s Republic of China

**Keywords:** Protein binding pockets prediction, Transformer encoder, Protein structural classification, Transfer learning

## Abstract

**Background:**

Previous deep learning methods for predicting protein binding pockets mainly employed 3D convolution, yet an abundance of convolution operations may lead the model to excessively prioritize local information, thus overlooking global information. Moreover, it is essential for us to account for the influence of diverse protein folding structural classes. Because proteins classified differently structurally exhibit varying biological functions, whereas those within the same structural class share similar functional attributes.

**Results:**

We proposed LVPocket, a novel method that synergistically captures both local and global information of protein structure through the integration of Transformer encoders, which help the model achieve better performance in binding pockets prediction. And then we tailored prediction models for data of four distinct structural classes of proteins using the transfer learning. The four fine-tuned models were trained on the baseline LVPocket model which was trained on the sc-PDB dataset. LVPocket exhibits superior performance on three independent datasets compared to current state-of-the-art methods. Additionally, the fine-tuned model outperforms the baseline model in terms of performance.

**Scientific contribution:**

We present a novel model structure for predicting protein binding pockets that provides a solution for relying on extensive convolutional computation while neglecting global information about protein structures. Furthermore, we tackle the impact of different protein folding structures on binding pocket prediction tasks through the application of transfer learning methods.

**Graphical Abstract:**

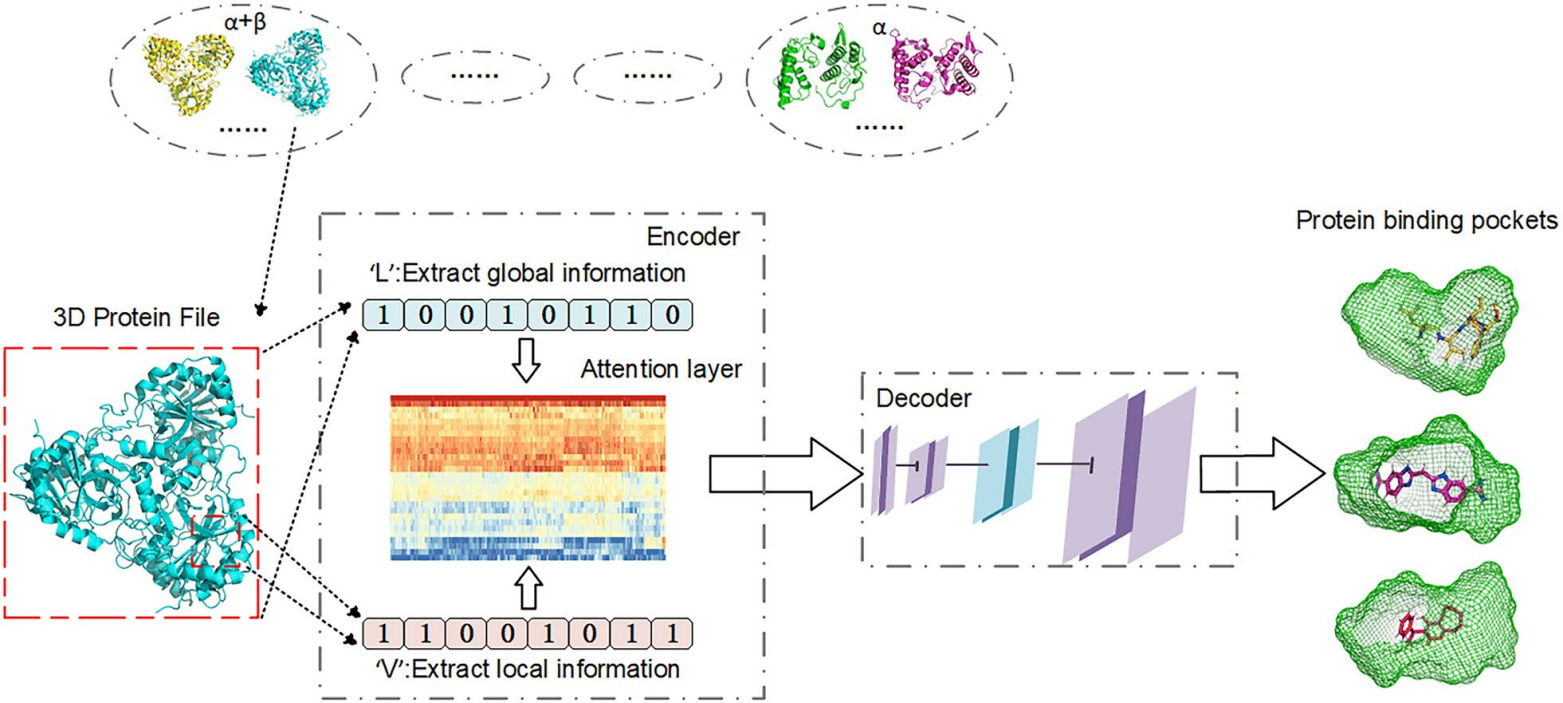

**Supplementary Information:**

The online version contains supplementary material available at 10.1186/s13321-024-00871-8.

## Introduction

Proteins are essential components of human cells and are involved in various biological processes within the organism. They play critical roles, such as facilitating the transportation of substances, modulating the immune system, catalyzing reactions, and regulating physiological processes. The identification of protein binding pockets is crucial for guiding drug design, protein function research. The critical step in Traditional Structure-Based Drug Design (SBDD) is to identify potential drug-binding pockets on the target protein and determine the amino acids that constitute these binding pockets [1]. Once the protein drug-binding pockets are identified, specific new small molecules can be designed and generated, thereby guiding and accelerating the drug design process.

The complex spatial configuration resulting from protein folding leads to an uneven protein surface, which in turn gives rise to the formation of cavities. These cavities are often the sites where drugs bind to proteins [2]. Protein binding pockets are cavities located on the protein's surface or interior, which can bind specifically to ligands. In addition, some protein binding pockets have druggability and play a crucial functional role. The amino acid residues around the binding pocket determine its shape, position, physicochemical properties, and functions [2].

Traditional methods for detecting binding pockets encompass geometry-based, energy-based, and template-based techniques. Geometry-based methods typically detect surface pockets in proteins using their 3D structure and rank them based on binding ability. Fpocket [3] is a geometry-based algorithm that utilizes Voronoi tessellation and alpha spheres clustering to detect protein pockets. The ConCavity [4], CriticalFinder [5] and POCKET [6] are also the classical geometry-based methods. The FTSite [7] is a successful energy-based methods that places 16 different probes on the protein grid and clusters them to predict binding sites. The Q-SiteFinder [8], AutoSite [9], EASYMIFs, SITEHOUND [10], SiteMap [11] are also the successful energy-based methods. FINDSITE [12] is a successful template-based method, identifying template proteins that bind to ligands in the PDB database and overlaying the template onto target proteins to ascertain binding sites. The LBias [13], and LIBRA [14] are other successful template-based methods.

In recent years, the advancement of artificial intelligence technology has led to the emergence of numerous new prediction methods for protein binding pockets, leveraging machine learning and deep learning. P2Rank [15] is a machine learning-based method for predicting binding sites from protein structures. P2Rank utilizes a random forest classifier to infer the coordination of local chemical neighborhoods near protein surfaces. DeepSite [16] is a deep learning method which is based on 3D convolutional neural networks. Kalasanty [17], a deep learning prediction method, is built upon the U-Net [18] architecture and utilizes a 3D image segmentation method for predicting protein pockets. PUResNet [19] is another deep learning method for predicting protein binding pockets, constructing a prediction model by integrating ResNet [20] and U-Net. DeepSurf [21] is a surface-based deep learning approach that predicts protein binding pockets by combining surface-based representations. PointSite [22], a point cloud segmentation tool, identifies protein binding pockets using a deep learning method that leverages the local connectivity of atoms within the protein. DeepPocket [23] combines geometric structure method with deep learning method. It utilizes 3D convolutional neural networks for the rescoring of pockets identified by Fpocket and further segments these identified cavities on the protein surface.

While protein binding pockets reside within the local structural domains of proteins, their characteristics are also influenced by the global protein structure. Thus, deep learning models should not focus solely on local information during feature learning but should also consider global information. These above approaches utilized 3D grids to represent protein structures and employed numerous 3D convolution operations for feature learning, which neglected the global information of the protein data. It is widely acknowledged that excessive use of convolutional computations can result in the model learning features that are overly concentrated and limited to specific locations. In response, we proposed a novel method which focused on reinforcing the learning of global information within protein 3D structure data. In our model, we incorporate V-Net [24], residual connections and Transformer encoder to concurrently capture both local and global information from protein data.

**Fig. 1:**
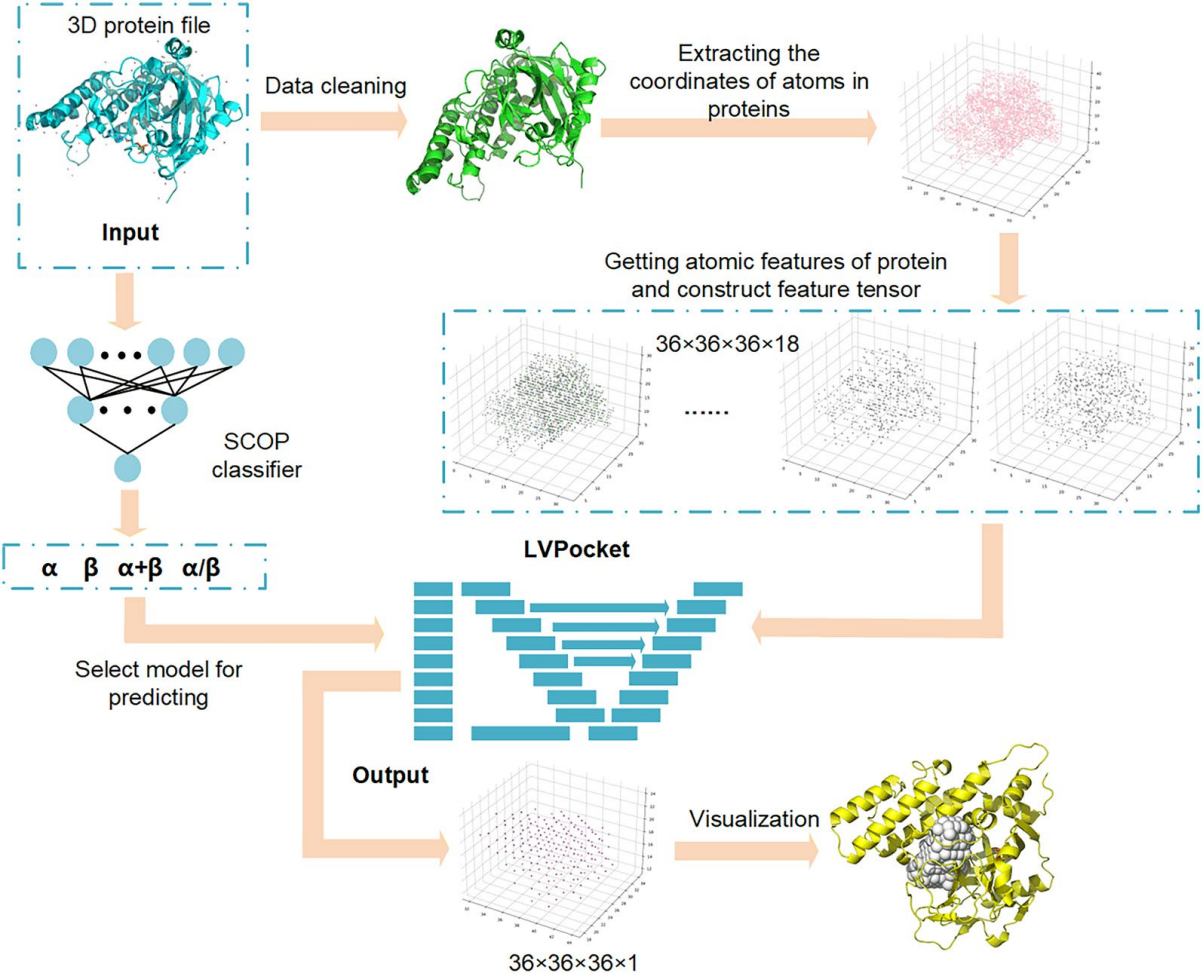
The workflow of our work. The input file consists of the 3D protein structure, followed by a data cleaning process. Subsequently, we extract the atomic coordinates from the protein and construct feature tensors comprising 18 attributes derived from these coordinates. Following this, we use a classifier to determine protein structure classification and then select a fine-tuned model or a baseline model based on the protein structure, which then receives the feature tensor of protein as input. Lastly, the model generates the predicted binding pockets

The 3D structure of proteins is widely recognized for its complexity, exhibiting significant dissimilarities among various protein structures. From a biological perspective, structural classification is crucial for comprehending the fundamental principles governing protein structure, function, and evolution [25]. Moreover, structure classification provides a valuable source of data for diverse analyses. However, in previous studies on protein pocket prediction, the impact of different structure classes formed by protein folding was not taken into account. Therefore, our study employs transfer learning to fine-tune models for different categories of protein structures. Our approach involves protein structure classification using the SCOP (Structural Classification of Proteins) database [26], constructing a protein binding pocket prediction model, and fine-tuning individual pocket prediction models for each structural categories by employing distinct parameters. Simultaneously, we have developed a protein structural classifier to assist people in identifying the structural classification of proteins. Figure 1 shows the workflow of LVPocket prediction.

## Methods

### Dataset preparation

The training dataset for our study was derived from the sc-PDB database [27], which includes the data of proteins, ligands, and pockets. The binding pockets are represented by 3D pocket shapes generated using VolSite [28]. The sc-PDB dataset (v.2017) consists of 17,594 protein–ligand complexes, corresponding to 16,612 protein structures and 5540 UniProt IDs. According to SCOP database, we classify the proteins into four groups based on the type of protein structure: (1) proteins that are predominantly alpha-helical (all α), (2) proteins containing predominantly beta-strands (all β), (3) proteins with alternating alpha-helices and beta-strands (α + β), (4) proteins with segregated alpha-helices and beta-strands (α/β), and the detail statistics are shown in Table 1. The Refined, SC6K and KV3K dataset are the test datasets which is to evaluate the generalization ability of our model. It is noteworthy that the protein binding pockets in these three test datasets were all generated using different tools, but the same parameters were based on ligands. Which could well verify the scalability of our model. The introduction is as follows:

- Refined: It derived from DeepPocket, a compilation of Refined subsets from v2007, v2013, v2015, and v2016 from PDBbind database [29]. It contains 2793 protein–ligand complexes and the protein binding pockets were generated by the VolSite. We classified the proteins into four classes based on their structure, as detailed in Table 1.
- SC6K: It also obtained from DeepPocket, which consists of 6285 protein–ligand complexes from the PDB (Protein Data Bank) database [30] between January 1, 2018, and February 28, 2020. The protein binding pockets in this dataset are generated by the IChem Toolkit [31]. The specifics of protein structural classification are elucidated in Table 1.
- KV3K: It was constructed by ourselves and curated directly from the PDB database, up until April 1, 2023. Then, we filtered the collected dataset according to the following criteria: (1) Removed duplicate data from the training dataset sc-PDB and other test datasets; (2) Eliminated complex polymeric proteins which contain more than 7 chains; (3) Excluded proteins that had read errors detected by OpenBabel [32]; (4) Removed proteins for which a ligand could not be parsed by KVFinder Toolkit [33]. Subsequently, we utilized the KVFinder Toolkit to generate protein binding pockets, employing the same filters and site selection algorithm as those used with the sc-PDB dataset. The dataset comprises 3134 protein–ligand complexes, and detailed structural classification statistics are presented in Table 1.

**Table 1:** The classification statistics of SCOP in sc-PDB, Refined, SC6K and KV3K datasets

	α	β	α and β	α or β	SCOP classify	Original
scPDB	2061	2133	3847	5281	13,322	17,594
Refined	271	666	615	765	2472	2793
SC6K	339	214	497	877	1927	6285
KV3K	668	438	592	612	2310	3134

To prevent data leakage, we removed the same protein data from the sc-PDB dataset as the above three test datasets. Additionally, due to a reading error in Open Babel, certain proteins were discarded, the PDB ID of the removed protein can be found in Supplementary Information.

Finally, 15,860 protein structures, aligning with 5473 UniProt entries, were employed for the training phase. Then we artificially select 1/10 data as the internal validation set during the training process and make sure all structures of a single protein must be in this set. This setup was necessary to avoid data leakage [17].

### Data processing

Prior to inputting the data into the model, we conducted operations on the 3D structural protein files, including the removal of water molecules and fragmented small molecules [23] to obtain pure protein structure data by using the Biopython library [34].

After cleaning the protein file, the prepared data undergoes tfbio program [35] to extract features such as atomic type and atomic property. Table 2 presents the nine atomic type features utilized in this study, which include boron, carbon, nitrogen, oxygen, phosphorus, sulphur, selenium, halogen, and metal atoms, and the detailed description of the nine atomic property features, namely hybridization, hydrophobicity, partial charge, heteroatoms, non-hydrogens, acceptor, aromatic, donor, and ring. The 18 atomic features were used to depict a protein by the tfbio program. To acquire the atomic features of a protein in 3D space, the positional coordinates of the atoms within the protein need to be obtained. These coordinates are represented by a two-dimensional array of size N*3, where N represents the number of atoms in the 3D protein file, and 3 represents the three-dimensional coordinates (x, y, z) of the atom. After determining the atom coordinates within the protein, the 3D protein structure was regarded as a 3D grid with dimensions of 36 × 36 × 36 × 18. A 3D grid measuring 36 × 36 × 36 was positioned at the center of the protein, with a distance of 70 Å in each direction. The grid representation of protein is the same as Kalasanty and PUResNet. In this 36 × 36 × 36 grid, each atom is assigned a unique value according to its properties at the corresponding voxel, while non-atomic voxels are set to 0. Consequently, the 3D protein structure is represented by a tensor of size 36 × 36 × 36 × 18, where each 36 × 36 × 36 3D tensor corresponds to an atomic feature. The 3D file of the protein binding pockets was represented using a same-sized 3D grid. For each voxel in the grid, a value of 1 was assigned if it belonged to a pocket, otherwise it was assigned 0.

**Table 2:** The nine atomic type features and nine atomic property features of proteins

Atom type	Description	Property	Description
B	Boron atom	Hybridization	The atom’s hybridization in the protein
C	Carbon atom	Hydrophobic	The hydrophobicity and hydrophilicity of an atom
N	Nitrogen atom	Partial charge	The partial charge of an atom
O	Oxygen atom	Heteroatoms	The number of heteroatoms attached to an atom
P	Phosphorus atom	Non-hydrogens	The number of non-hydrogens attached to an atom
S	Sulphur atom	Acceptor	The non-acceptor and acceptor atoms in the protein
Se	Selenium atom	Aromatic	The aliphatic and aromatic atoms in the protein
F、Cl、Br、I	Halogen atom	Donor	The donor and non-donor atoms in the protein
Atomic number: 3,4,11,12,13,19 ~ 32,37 ~ 51,55 ~ 84,87 ~ 104	Metal atom	Ring	The atoms in and not in ring in the protein

Finally, the processed 3D protein gird data will be input to the training model, and the 3D grid data of binding pockets will be input to the model as labels.

### Model Structure

LVPocket is constructed on the foundational framework of the V-Net model, incorporating concepts from residual connections and the Transformer [36] model. The V-Net model offers an end-to-end 3D image segmentation approach that effectively address the significant imbalance between foreground and background voxels. In this context, we utilized the V-Net model to predict protein binding pockets. In order to mitigate the loss of original data features caused by an excessive number of convolutional layers, we have introduced residual connections. The intermediate tensor generated during encoding is passed from the encoder to the decoder through the residual connection, enabling the decoder to more refer to the input information during decoding.

The initial V-Net model predominantly focused on localized feature extraction from the data. Consequently, we enhanced the encoder section by introducing an additional pathway involving a limited number of convolution operations. This augmentation aimed to extract global information from the original data. Recognized for its exceptional capacity to directly capture global data information, the Transformer model surpasses conventional convolution operations. Therefore, we added two Former layers in our model, which combined 3D convolution with the encoder of transformer to maximize the extraction of global protein feature information. To balance data information concentration and computational complexity, we placed two Former layers in the middle position of the two encoder paths.

Diverging from the original Transformer encoder, our Former layer integrates a 3D convolutional layer with a kernel size of 1 × 1 × 1 to capture comprehensive global information. Since input tensor consist of 18 36 × 36 × 36 grids, we flattened the 36 × 36 × 36 tensor into one-dimensional form to facilitate the computation of multi-head attention. The formulas of multi-head attention are as follow:

1 $$Attention(Q, K, V)=\text{softmax}(\frac{\text{Q}{K}^{T}}{\sqrt{{d}_{k}}})V$$

2 $${head}_{i}=\text{Attention}(\text{X}{Q}_{i},\text{ X}{K}_{i},\text{X}{V}_{i})$$

3 $$MultiHead\left(X\right)=\text{Concat}\left({head}_{1, }\dots \dots ,{head}_{i}\right)$$

The *X* is the input tensor, the *Q, K, V* represent the queries, keys and values of the input tensor, and the *d*_*k*_ represents the dimension of the *Q, K, V*. The *head*_*i*_ represents the *i-th* attention head.

**Fig. 2:**
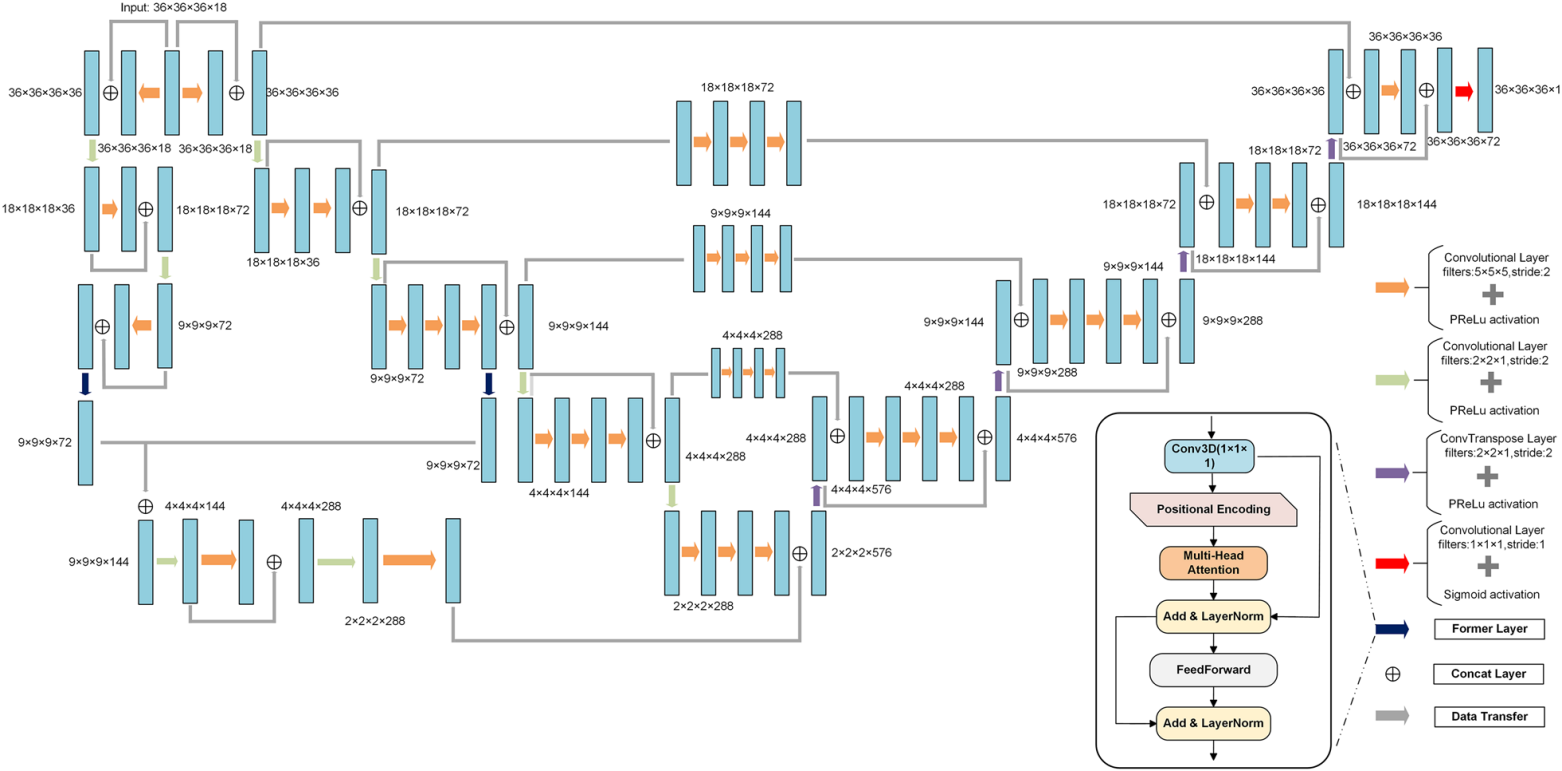
The detailed model structure of LVPocket. The orange arrow signifies a 3D convolution operation with a kernel size of 5 × 5 × 5 and a prelu activation calculation. The green arrow represents a 3D convolution operation with a kernel size of 2 × 2 × 1 and a prelu activation calculation. The red arrow represents a 3D convolution operation with a kernel size of 1 × 1 × 1 and a sigmoid activation calculation. The purple arrow denotes a 3D convtranspose operation with a prelu activation calculation, and the dark blue arrow indicates the Former layer. The blue rectangle represents the data block, and the grey arrow represents the data transfer operation

LVPocket consists of two primary components: an encoder and a decoder, as illustrated in Fig. 2, with a detailed structure. Firstly, the encoder section consists of two distinct pathways. The ‘L’ pathway on the left extracts global information by integrating former layer and 3D convolution operations. Conversely, the left section of the V-Net encoder focuses on capturing local protein information using abundant 3D convolution operations. Furthermore, an information exchange takes place between these two pathways. After undergoing 8 convolutions in the encoder part of the V-Net and the former layer, the data is transmitted to the ‘L’ pathway. Then it is concatenated with the tensor passing though the former layer of the ‘L’ pathway. Subsequently, the ultimate output from the two encoder pathways is concatenated and used as the input for the decoder. These two encoder pathways enable the model to concurrently integrate global and local information pertaining the 3D protein structure.

### Model training

We employ the Dice Loss function, derived from the Dice Coefficient, as the loss function [37]. Formula (4) illustrates the relationship between them. The Dice Coefficient is a statistic used to assess the similarity between two samples by quantifying their overlap, with with a value range of [0,1]. Formula (5) presents its definition, where *|X ∩ Y|* represents the intersection of sets X and Y, and |*X*| and |*Y*| represent the number of elements in the respective sets. In the task of predicting protein binding pockets, |*X* | and *|Y|* correspond to the actual and predicted protein pockets, respectively. Formula (6) presents the detailed equation for the Dice Loss, in which y represents the predicted value of the model, *t* represents the actual value, and *i*, *j*, and *k* represent the three-dimensional coordinates of the atom. The interference factor is represented by *ɛ*.

4 $$Dice \, Loss=1- Dice \, Coefficient.$$

5 $$Dice \,  Coefficient=\frac{2 | X\cap Y |}{\left| X \right|+| Y |}.$$

6 $$C\left(y,t\right)=1-\frac{2{\sum }_{i,j,k}\left({y}_{i,j,k}\cdot {t}_{i,j,k}\right)+\upvarepsilon }{{\sum }_{i,j,k}\left({y}_{i,j,k}+{t}_{i,j,k}\right)+\upvarepsilon }.$$

Throughout the training process of the baseline model, we employed the breakpoint continuation training method. The loss value on the internal validation set served as an indicator for the selection of hyperparameters for the model. We fine-tuned the model by leveraging the pre-trained baseline model and incorporating SCOP classification data from the sc-PDB dataset.

When training the model, we used an NVIDIA A100 GPU. The baseline model underwent training for 1800 epochs, while the four SCOP models (all α, all β, α + β, α/β) were fine-tuned for 100, 200, 400, and 500 epochs, respectively. The batch size was set to 10, with each computation round taking 105 s.

### Evaluation metrics

Three main metrics are used to evaluate the performance of protein binding pocket detection algorithms. These metrics evaluate the algorithm's ability in detecting the position and shape of the binding pockets. These metrics include:

- Distance to the center of the binding pocket (DCC) [17, 19, 23]. It is the distance between the center of the predicted binding pocket and the center of the real protein binding pocket. If the distance is less than 4 Å, it will be determined to be a successfully predicted pocket. We calculate the success rate of the entire prediction dataset by DCC. This is shown in Eq. (7).
  7 $$Success \, Rate= \frac{Number \, of \, pocket \, having \, DCC\le 4\text{ \AA }}{Total \, number \, of \, pockets}$$
- Discretized volume overlap (DVO) [17, 19, 23]. It is the ratio between the volumetric intersection between the predicted (*V*_*pbs*_) and actual binding site (*V*_*abs*_) to their union. The volume is the set of voxels with a value of 1. We calculate it by the Jaccard index formula, as shown in Eq. (8).
  8 $$DVO= \frac{{V}_{pbs}\cap {V}_{abs}}{{V}_{pbs} \cup {V}_{abs}}$$
- Distance to any atom of the ligand (DCA) [23]. It is defined as the minimum distance between the predicted pocket center and any atom within the ligand. Predictions with DCA ≤ 4 Å are considered successful.

### The SCOP classifier

The model structure of the classifier, along with evaluation performance information, can be found in the Additional file.

## Results and discussion

### The comparison between baseline model and other methods

In order to comprehensively evaluate the performance of the model, we compared baseline model with three deep learning methods Kalasanty, PUResNet, and DeepPocket using the three aforementioned metrics on the Refined, SC6K and KV3K datasets. We employed open-source code and trained model files from these completed methods for predicting protein pockets. We inputted the protein data from the three test datasets into different models for prediction, and then compared their predicted pockets with the actual binding pockets in the test datasets.

We conducted a comparison of DCC success rate and DCA success rate for both the methods on the Refined, SC6K and KV3K datasets, as detailed in Table 3. When DCC ≤ 4 Å, LVPocket attains the highest success rate among the three datasets. When DCA ≤ 4 Å, LVPocket demonstrates the highest success rate on the Refined and KV3K datasets. On the SC6K dataset, its success rate closely approaches that of DeepPocket, outperforming other methods. DVO values were calculated for predicted pockets with DCC ≤ 4 Å. Table 4 displays the number of binding pockets predicted by different methods when DVO ≥ 0.4. A higher DVO value indicates a greater similarity between the predicted pocket shape and the actual pocket shape.

**Table 3:** The success rate of LVPocket and other methods when DCC, DCA ≤ 4 Å

	Refined		SC6K		KV3K	
	DCC (%)	DCA (%)	DCC (%)	DCA (%)	DCC (%)	DCA (%)
Kalasanty	58.06	71.75	45.70	73.51	63.45	71.76
PUResNet	60.56	69.93	61.93	78.07	64.95	72.87
DeepPocket	70.08	77.48	56.25	79.28	66.60	74.53
LVPocket	70.27	77.75	62.60	78.11	71.94	77.28

**Table 4:** The number of the predicted pockets with different methods when DVO ≥ 0.4

	Refined	SC6K	KV3K
Kalasanty	24	92	473
PUResNet	922	1347	904
DeepPocket	315	284	634
LVPocket	1009	1380	870

In general, the predictions of LVPocket are closer to real protein binding pockets in comparison to other methods. We believe that LVPocket captures more globally useful information through the introduction of another encoder path, and the Former plays an important role.

### The ablation experiment and visualization comparison with V-Net

In our initial endeavors, we utilized the V-Net model for the training of 3D structural protein data. Nevertheless, the model's performance proved to be unsatisfactory. A thorough examination of the encoder section revealed an excessive number of convolutional layers. This configuration caused the model to predominantly emphasize local information within the 3D protein structure, neglecting global information. To address this issue, we introduced an additional pathway to the encoder, with the aim of enhancing its ability to capture global information. Ultimately, we integrated a novel encoding pathway into the V-Net model's encoder section, resulting in a model structure shaped like ‘LV.’ Subsequently, we drew inspiration from the Transformer model and gained insights into its advantages in extracting global information from data. We constructed a new layer, the Former layer, which is modified by the encoder of Transformer. To assess the effectiveness of the modifications to the V-Net, we conducted ablation experiments on the internal validation dataset, comparing them based on loss value, DCC, DCA, average DVO and predicted pockets count metrics. The internal validation dataset contains a total of 1586 entries data and the three models of V-Net, LV-Net(without Former layer) and LV-Net(with Former layer) predicted the number of binding pockets on the internal validation dataset to be 1460, 1520, 1583, which indicates the generation ability of LV-Net(with Former layer) in learning global information. The results of the ablation experiments demonstrated the effectiveness of our modifications and optimizations to the V-Net, as presented in Table 5.

**Table 5:** The results of ablation experiments

	V-Net	LV-Net (without Former layer)	LV-Net (with Former layer)
LOSS	0.34	0.32	0.31
DCC	0.73	0.74	0.77
DCA	0.83	0.85	0.88
Average DVO	0.42	0.43	0.44
Pockets Count	1460	1520	1583

**Fig. 3:**
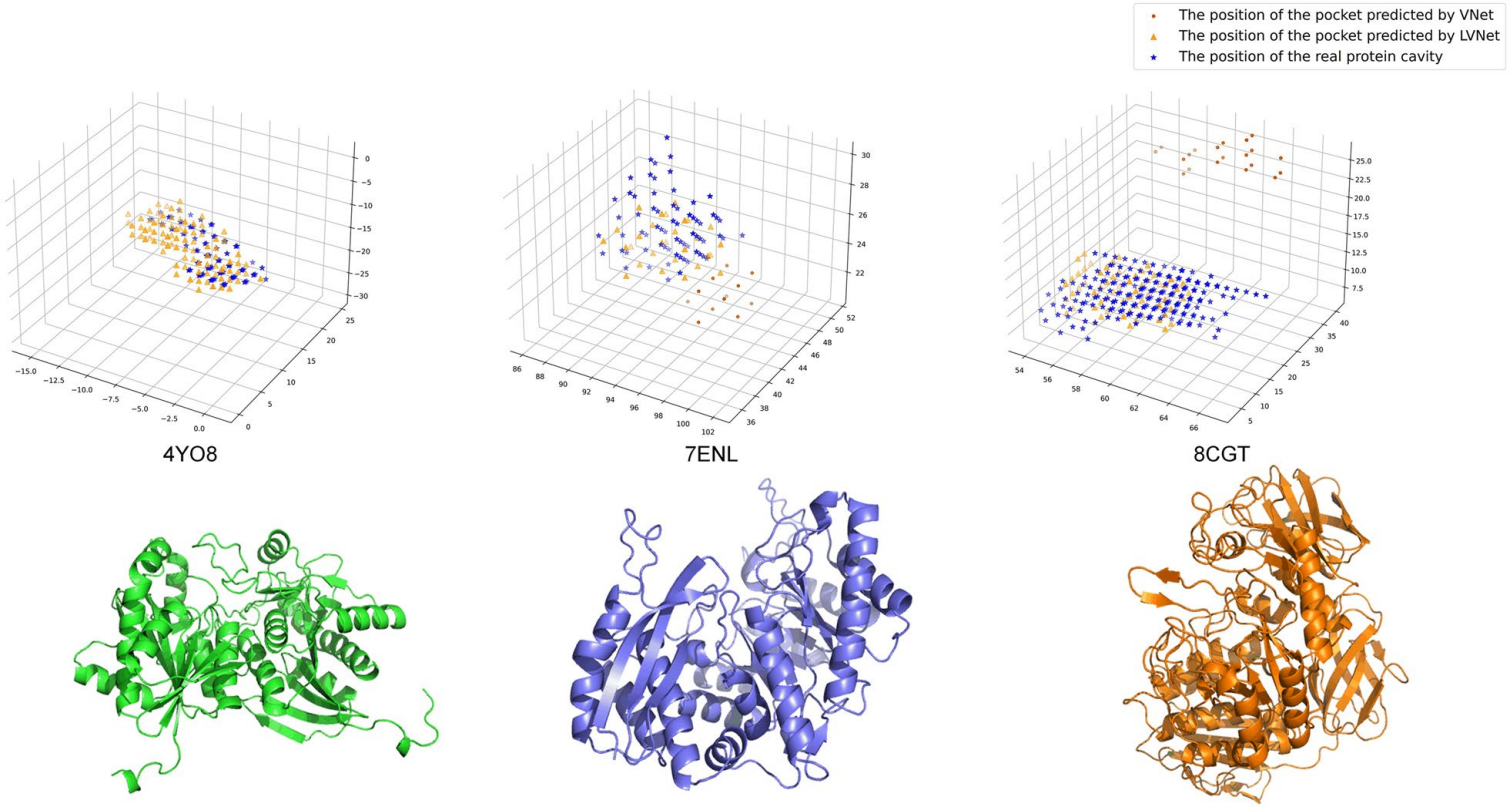
The comparison of the ability of dealing with complex proteins between LV-Net and V-NET. The blue pentagrams are the position of real protein binding pockets, the yellow triangles are the position of pocket predicted by LV-Net and the red dots are the position of pocket predicted by V-Net

Figure 3 provides a visualization comparison of binding pockets predicted by LV-Net (with Former layer) and V-Net on three complex proteins. LV-Net (with Former layer) demonstrates a comparative advantage when dealing with the complex protein structures. Such as the protein 4Y08 (PDB ID), 7ENL (PDB ID) and 8CGT (PDB ID), the predicted pocket of LVPocket is coincident with the real pockets, but the predicted pocket of V-Net is far from the real pocket.

### The comparison between baseline model and SCOP models

The SCOP models build upon the baseline model by fine-tuning the model parameters for SCOP data in order to increase prediction specificity. We performed SCOP classification on the Refined, SC6K and KV3K datasets, employing SCOP models to predict protein pockets accordingly, and compared the performance in identifying binding pockets with the baseline model on three test datasets.

The DCC success rate in all SCOP classifications are reported in Table 6, the fine-tuned model exhibits superior success rates to the baseline model when DCC is ≤ 4 Å. The DCC visual comparison of baseline model and other SCOP fine-tuned model are shown in Additional Fig. 3. Notably, the success rates of α/β classifications are inferior compared to other classes. We deduce that the reason for this is that the proteins in α/β class are more complex than others. We also calculated the DCA success rate metric on the baseline model and fine-tuning model as presented in Table 7. Similarly, the SCOP fine-tuned models surpassing the baseline model, and the DCA visual comparison of LVPocket and SCOP fine-tuned model are shown in Additional Fig. 4. Table 8 displays the number of the predicted pockets with baseline model and four fine-tuned models on the Refined, SC6K and KV3K datasets. It is evident that the performance of fine-tuned model has a certain improvement.

**Table 6:** When DCC ≤ 4 Å, the success rate of baseline model and fine-tuned model

	Refined		SC6K		KV3K	
	Base line (%)	Fine-tuned (%)	Base line (%)	Fine-tuned (%)	Base line (%)	Fine-tuned (%)
α	72.87	77.24	63.12	64.17	73.39	74.80
β	77.62	79.42	67.30	68.45	74.37	77.78
α and β	72.16	76.27	70.97	71.78	78.67	80.56
α or β	65.19	69.41	62.85	64.44	59.19	60.45

**Table 7:** When DCA ≤ 4 Å, the success rate of baseline model and fine-tuned model

	Refined		SC6K		KV3K	
	Base line (%)	Fine-tuned (%)	Base line (%)	Fine-tuned (%)	Base line (%)	Fine-tuned (%)
α	77.38	80.01	70.17	71.43	79.19	80.48
β	84.69	86.20	75.81	78.07	77.16	80.72
α and β	81.51	85.98	81.14	83.40	83.73	85.53
α or β	70.21	72.77	80.76	81.07	65.06	66.56

**Fig. 4:**
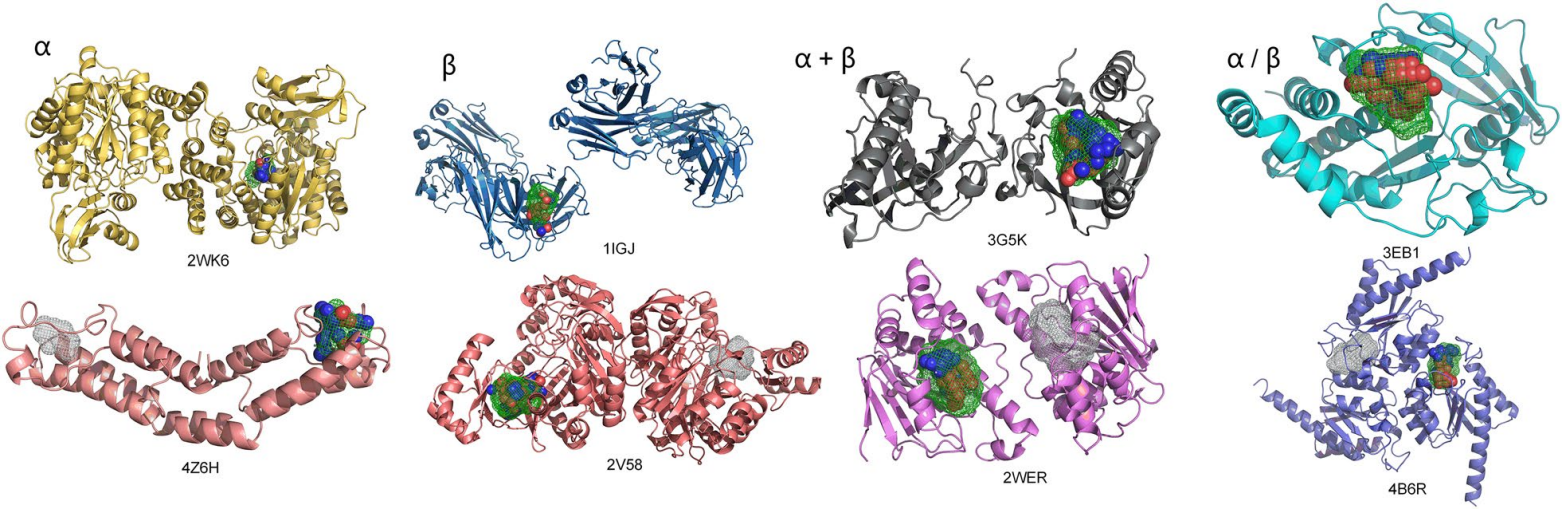
The visualization of the prediction pockets of baseline model and SCOP fine-tuned model. The green grid is the prediction pocket of fine-tuned model, the gray grid is the prediction pocket of baseline model and the red and blue spheres represent the real pocket in the protein

**Table 8:** The number of the predicted pockets with baseline model and four fine-tuned models when DVO ≥ 0.4

	Refined		SC6K		KV3K	
	Base line	Fine-tuned	Base line	Fine-tuned	Base line	Fine-tuned
α	111	120	92	95	186	188
β	313	319	54	60	90	93
α and β	280	306	137	142	180	182
α or β	172	210	155	168	114	122

In summary, a comparison with the baseline model demonstrating that after fine-tuning, the performance of the SCOP fine-tuned model significantly outperforms the baseline model. Notably, even in the most complex protein class α/β, fine-tuned model exhibits superior predictions compared to the baseline model. The experiment comparing the baseline model and SCOP models aims to demonstrate our idea that variations among different protein folding structural classes affect protein binding pocket prediction. And we believe that our idea can also be applied in other concurrent methods.

### Application of transfer learning strategy

Our study stands out from previous approaches by considering the influence of protein secondary structure classification on dealing with the protein data. The challenge in learning model features is intricately linked to the complexity inherent in protein structures. To address this, we classified proteins into four SCOP classes, enabling the model to discern distinctive features within each class. This approach significantly augments the model's specificity. Our specific methodology involves fine-tuning a pre-trained baseline model using classified data. This process allows the model to focus on features associated with a specific structural class, leading to improved prediction performance of model in that structural class. In some cases, the baseline model failed to accurately predict the positions of specific protein binding pockets, whereas the SCOP fine-tuned model excelled. As depicted in Fig. 4, for proteins 2WK6 (PDB ID), 1IGJ (PDB ID), 3G5K (PDB ID), and 3EB1 (PDB ID), the baseline model inaccurately predicted the binding pocket, while the fine-tuned model exhibited precise predictions. For proteins 4Z6H (PDB ID), 2V58 (PDB ID), 2WER (PDB ID), and 4B6R (PDB ID), although the baseline model generated a prediction pocket, it deviated significantly from the real pocket.

Considering this the method of transfer learning can be extended to protein data with other classification standards. For instance, in the task of predicting binding pockets for protein kinases (PK), the model can undergo fine-tuning using protein data, resulting in a highly specific pocket prediction model tailored for PK proteins.

### Analysis of protein multi-pocket prediction results

**Fig. 5:**
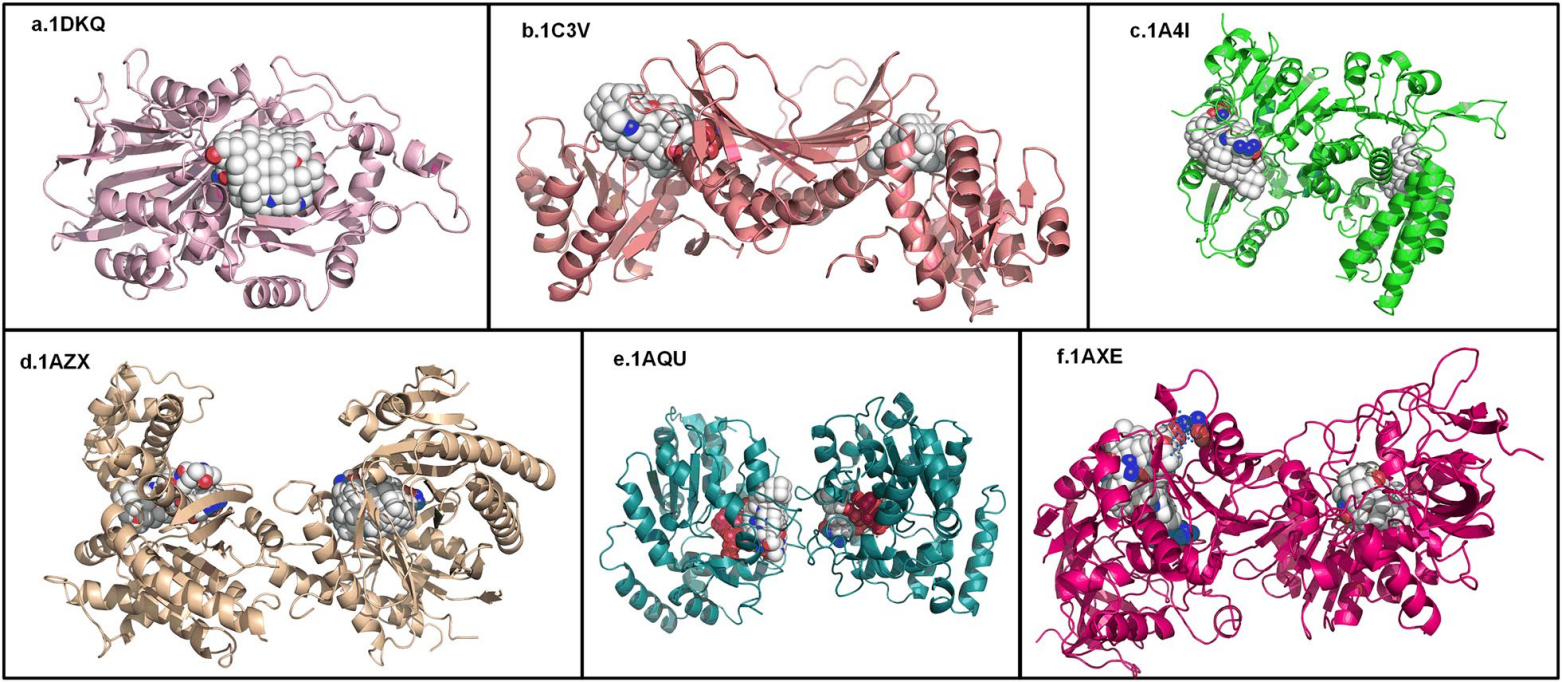
The visualization of multi-prediction pockets of LVPocket. The white spheres are the predicted binding pockets, and the red and blue spheres are the real binding pockets

LVPocket possesses the ability to generate either a single or multiple protein pockets for a given protein. Figure 5-a illustrates accurate prediction by LVPocket of a single binding pocket for the 1DKQ (PDB ID), aligning with the real pocket. In certain instances, LVPocket predicts two binding pockets for a protein, with one aligning accurately and the other not. However, we posited that the non-coinciding binding pocket may signify an undiscovered novel binding pocket for the protein. This speculation arises from the acknowledgment that presently identified protein binding pockets are not exhaustive, as depicted in Fig. 5-b, Fig. 5-c, Fig. 5-d. Naturally, there are instances where both of LVPocket's predicted pockets align perfectly with the two real pockets, as shown in Fig. 5-e, Fig. 5-f. In scenarios where LVPocket predicts multiple protein binding pockets, the presence of at least one predicted pocket coinciding with the real pocket holds greater significance. This is because if one predicted pocket aligns with the real pocket, it serves as evidence of LVPocket's could accurately deal with this protein. Furthermore, the additional binding pockets predicted by LVPocket provide valuable reference points.

## Conclusion

In this study, we introduced a innovative model structure for predicting protein binding pockets, incorporating the transfer learning to enhance the prediction performance on different protein structure. Comparisons with similar existing methods have demonstrated that heightened focus on global information in protein structure data enhances the predictive performance of the model. Our SCOP fine-tuned model exhibits significant improvements compared to the baseline model. While our model adeptly handles the 3D structures of intricate proteins, there remains room for refinement in predicting binding pockets, particularly for complex polymeric proteins. Based on our study, we consider it is crucial to explore methods for enhancing the accuracy of predictions for complex polymeric proteins. Currently, our predicted protein binding pockets only include their 3D spatial coordinates. Our future research aims to identify the types of atoms suitable for placement at each position within the protein binding pocket.

### Supplementary Information

Additional file 1. The figure of model structure of SCOP classifier.

Additional file 2. The figure of workflow of the SCOP classifier.

Additional file 3. The table of the description of the protein secondary structure features.

Additional file 4. The table of test metrics of SCOP classifier on the independent dataset 25-1.

Additional file 5. The table of test metrics of SCOP classifier on the independent dataset 640-1.

Additional file 6. The figure of visualization of DCC success rate

Additional file 7. The figure of visualization of DCA success rate

Additional file 8. The PDB ID of proteins which is reading error in Open Babel.

## Data Availability

All the codes related to this method are publicly available at https://github.com/ZRF-ZRF/LVpocket. The training dataset and KV3K dataset are available at: 10.5281/zenodo.10633986. The pretrained model files are available at 10.5281/zenodo.10633690.

## Abbreviations

SCOP: Structural classification of proteins
SBDD: Structure-Based Drug Design
DCC: Distance to the center of the binding pocket
DVO: Discretized volume overlap
DCA: Distance to any atom of the ligand
