# Supplementary material for: LVPocket: integrated 3D global-local information to protein binding pockets prediction with transfer learning of protein structure classification
Source: J Cheminform. 2024 Jul 7;16:79. doi: 10.1186/s13321-024-00871-8 (PMC11229186; doi:10.1186/s13321-024-00871-8)
Supplement: Supplementary file 1 — Additional file 1. The figure of model structure of SCOP classifier. [file 13321_2024_871_MOESM1_ESM.docx]

**Additional Files**

**LVPocket: Integrated 3D Global-local Information to Protein Binding Pockets Prediction with Transfer Learning of Protein Structure Classification**

Ruifeng Zhou^1^, Jing Fan^1^, Sishu Li, Wenjie Zeng^1^, Yilun Chen^1^, Xiaoshan Zheng^1^, Hongyang Chen^2,*^, Jun Liao^1,2,*^

^1^ The Research Center for Graph Computing, Zhejiang Lab, Hangzhou 311100, China.

^2^ China Pharmaceutical University School of Science, Nanjing 211198, China.

**Corresponding authors**

**Hongyang Chen: E-mail:** hongyang@zhejianglab.com

**Jun Liao: E-mail:** liaojun@cpu.edu.cn

**Contents**

[Additional file 1 3](#_Toc159317282)

[Additional file 2 3](#_Toc159317283)

[Additional file 3 4](#_Toc159317284)

[Additional file 4 5](#_Toc159317285)

[Additional file 5 5](#_Toc159317286)

[Additional file 6. 6](#_Toc159317287)

[Additional file 7. 7](#_Toc159317288)

[Reference 8](#_Toc159317289)


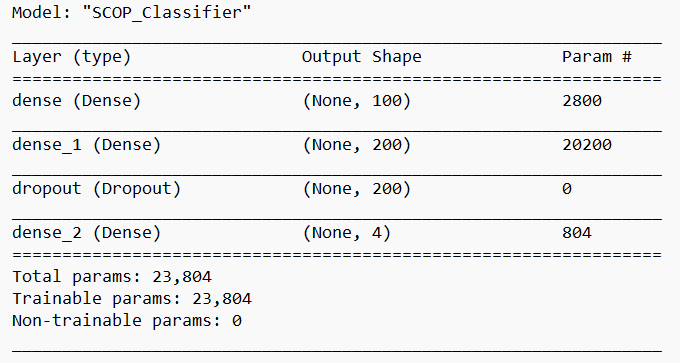


Additional file 1**.** The model structure of SCOP classifier.





# Reference

1. McGuffin, L. J.; Bryson, K.; Jones, D. T., The PSIPRED protein structure prediction server. *Bioinformatics* **2000**, 16, 404-405.

2. Kurgan, L.; Cios, K.; Chen, K., SCPRED: Accurate prediction of protein structural class for sequences of twilight-zone similarity with predicting sequences. *Bmc Bioinformatics* **2008**, 9.

3. Zhang, L. C.; Zhao, X. Q.; Kong, L., A protein structural class prediction method based on novel features. *Biochimie* **2013**, 95, 1741-1744.

4. Liu, T. A.; Jia, C. Z., A high-accuracy protein structural class prediction algorithm using predicted secondary structural information. *J Theor Biol* **2010**, 267, 272-275.

5. Zhang, L. C.; Zhao, X. Q.; Kong, L.; Liu, S. X., A novel predictor for protein structural class based on integrated information of the secondary structure sequence. *Biochimie* **2014**, 103, 131-136.
