# Supplementary material for: LVPocket: integrated 3D global-local information to protein binding pockets prediction with transfer learning of protein structure classification
Source: J Cheminform. 2024 Jul 7;16:79. doi: 10.1186/s13321-024-00871-8 (PMC11229186; doi:10.1186/s13321-024-00871-8)
Supplement: Supplementary file 2 — Additional file 2. The figure of workflow of the SCOP classifier. [file 13321_2024_871_MOESM2_ESM.docx]

Additional file 2**.** The workflow of the SCOP classifier. The protein file is the input data, and get the protein sequence from the file. Then we utilize PSIPRED ^1^ to extract the secondary structure features as input for the classifier. Finally, the classifier gives the classified results of the input protein.
