# Supplementary material for: LVPocket: integrated 3D global-local information to protein binding pockets prediction with transfer learning of protein structure classification
Source: J Cheminform. 2024 Jul 7;16:79. doi: 10.1186/s13321-024-00871-8 (PMC11229186; doi:10.1186/s13321-024-00871-8)
Supplement: Supplementary file 3 — Additional file 3. The table of the description of the protein secondary structure features. [file 13321_2024_871_MOESM3_ESM.docx]

Additional file 3**.** The description of the protein secondary structure features

| Feature | Description |
| --- | --- |
| P(H), P(E)^2^ | The content of H and E in SSS |
| CMVH, CMVE^2^ | The reflect the spatial arrangements of H and E in the SSS |
| MHN, MEN^3^ | The normalized lengths of the longest α-helices and β-strands in the SSS |
| NAvgH, NAvgE^3^ | The average lengths of the α-helices and β-strands in the SSS |
| NPNE, NAPNE^4^ | The normalized parallel and anti-parallel β-strands in the SSS. |
| MaxDHEN, MaxDEHN^5^ | Maximum distance normalized between adjacent α-helices and β-sheets fragments in the SSS |
| TEE, THH, THE, TEH^5^ | The fraction of HH, EE, EH, HE in HES were proposed to represent the level of aggregation about α-helices and β-strands. |
| ITHE, ITHH, ITEE, ITEH^5^ | Since the number of HH and EE in IHES could reflect the level of aggregation about a-helices and b-strands as well as the composition elements HE and EH in IHES, ITHE and ITEH revealed that they formed either terminal end of a b-strand or an a-helix that folded into parallel b-sheets. Hence, the protein that include the higher ITHH or ITEE may be the a þ b class and the higher ITHE or ITEH may be the a/b class. |
| PHS, PES^2^ | The content of H and E in SS. |
| CMVHS, CMVES^2^ | The reflect the spatial arrangements of H, and E in the SS. |
| NCountH6, NCountH8^2^ | Normalized count of α-helix segments (including at least 6/8 residues). |
| NCountE5^2^ | Normalized count of β-strand segments (including at least 5 residues) |
