# Supplementary material for: LVPocket: integrated 3D global-local information to protein binding pockets prediction with transfer learning of protein structure classification
Source: J Cheminform. 2024 Jul 7;16:79. doi: 10.1186/s13321-024-00871-8 (PMC11229186; doi:10.1186/s13321-024-00871-8)
Supplement: Supplementary file 5 — Additional file 5. The table of test metrics of SCOP classifier on the independent dataset 640-1. [file 13321_2024_871_MOESM5_ESM.docx]

Additional file 5**.** The metrics of SCOP classifier on the independent dataset 640-1.

| **classification** | **count** | **precision** | **recall** | **f1-score** |
| --- | --- | --- | --- | --- |
| **α** | 138 | 0.91 | 0.81 | 0.86 |
| **β** | 154 | 0.81 | 0.83 | 0.82 |
| **α/β** | 177 | 0.84 | 0.81 | 0.83 |
| **α+β** | 171 | 0.66 | 0.73 | 0.69 |
|  | | | | |
| **accuracy** | 640 |  | | 0.79 |
