# Supplementary material for: LVPocket: integrated 3D global-local information to protein binding pockets prediction with transfer learning of protein structure classification
Source: J Cheminform. 2024 Jul 7;16:79. doi: 10.1186/s13321-024-00871-8 (PMC11229186; doi:10.1186/s13321-024-00871-8)
Supplement: Supplementary file 6 — Additional file 6. The figure of visualization of DCC success rate [file 13321_2024_871_MOESM6_ESM.docx]

Additional file 6. The success rate of different DCC of baseline model and SCOP fine-tuned model on the Refined, SC6K and KV3K datasets.
