# Supplementary material for: LVPocket: integrated 3D global-local information to protein binding pockets prediction with transfer learning of protein structure classification
Source: J Cheminform. 2024 Jul 7;16:79. doi: 10.1186/s13321-024-00871-8 (PMC11229186; doi:10.1186/s13321-024-00871-8)
Supplement: Supplementary file 7 — Additional file 7. The figure of visualization of DCA success rate [file 13321_2024_871_MOESM7_ESM.docx]

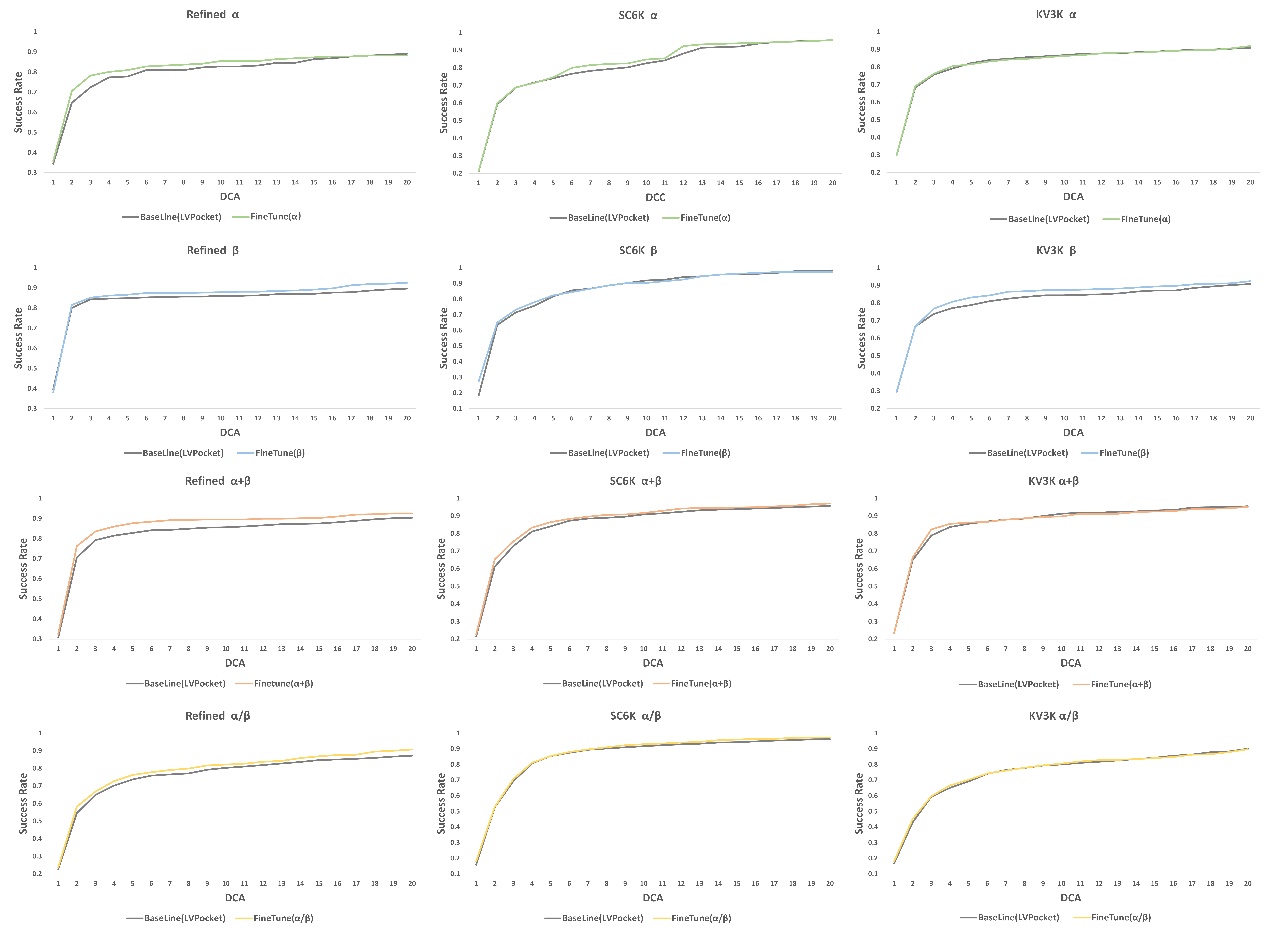


Additional file 7. The success rate of different DCA of baseline model and SCOP fine-tuned model on the Refined, SC6K and KV3K dataset
